# A Systematic Review and Meta-Analysis of the Global Seasonality of Norovirus

**DOI:** 10.1371/journal.pone.0075922

**Published:** 2013-10-02

**Authors:** Sharia M. Ahmed, Benjamin A. Lopman, Karen Levy

**Affiliations:** 1 Epidemiology Branch, Division of Viral Diseases, National Center for Immunization and Respiratory Diseases, Centers for Disease Control and Prevention, Atlanta, Georgia, United States of America; 2 Department of Environmental Health, Emory University, Atlanta, Georgia, United States of America; Northeastern University, United States of America

## Abstract

**Background:**

Noroviruses are the most common cause of acute gastroenteritis across all ages worldwide. These pathogens are generally understood to exhibit a wintertime seasonality, though a systematic assessment of seasonal patterns has not been conducted in the era of modern diagnostics.

**Methods:**

We conducted a systematic review of the Pubmed Medline database for articles published between 1997 and 2011 to identify and extract data from articles reporting on monthly counts of norovirus. We conducted a descriptive analysis to document seasonal patterns of norovirus disease, and we also constructed multivariate linear models to identify factors associated with the strength of norovirus seasonality.

**Results:**

The searched identified 293 unique articles, yielding 38 case and 29 outbreak data series. Within these data series, 52.7% of cases and 41.2% of outbreaks occurred in winter months, and 78.9% of cases and 71.0% of outbreaks occurred in cool months. Both case and outbreak studies showed an earlier peak in season-year 2002-03, but not in season-year 2006-07, years when new genogroup II type 4 variants emerged. For outbreaks, norovirus season strength was positively associated with average rainfall in the wettest month, and inversely associated with crude birth rate in both bivariate and multivariate analyses. For cases, none of the covariates examined was associated with season strength. When case and outbreaks were combined, average rainfall in the wettest month was positively associated with season strength.

**Conclusions:**

Norovirus is a wintertime phenomenon, at least in the temperate northern hemisphere where most data are available. Our results point to possible associations of season strength with rain in the wettest month and crude birth rate.

## Introduction

Noroviruses are the most common cause of sporadic cases and outbreaks of gastroenteritis across all age groups worldwide [1]–[3]. In the United States, norovirus is estimated to cause 21 million illnesses [2], 71,000 hospitalizations [4], and 800 deaths annually [2], [5]. In developed countries, outbreaks frequently occur in healthcare, long-term care, daycare, and school settings [6], and are associated with deaths among the elderly [7].

The winter seasonality of norovirus has long been recognized, and norovirus has been referred to as ‘*winter vomiting disease*’[8]. Understanding the relationship between climate and infectious diseases can help evaluate the impact of environmental drivers of infection risk and anticipate how global climate change will affect the distribution and spread of infectious diseases. For norovirus, climate change has the potential to impact seasonality by influencing transmissibility, host susceptibility, and the resistance of norovirus to environmental conditions [9].

Some studies have found associations between norovirus seasonality and climatic/weather phenomena. Rainfall has been highlighted as an important factor for norovirus seasonality, probably due to waterborne transmission of the virus [10]. Likewise, cool temperatures, low population immunity, and the emergence of new variants are associated with increased norovirus activity [11]. Of these predictors, changes in temperature had the greatest attributable risk for norovirus incidence in a long-term study of England and Wales [11]. Seasonal patterns of rotavirus, another gastrointestinal virus that peaks during winter months, have also been associated with weather factors such as temperature, relative humidity, and rainfall [12]–[17].

Factors related to human behavior, demographics, and host immunity have been put forth to explain increased incidence of norovirus in colder months. In particular, crowding and more time spent indoors are possible factors in increasing human-to-human transmission of respiratory viruses during winter [18]. For rotavirus, birth rates and local exposure rates (force of infection) have been suggested as key factors driving seasonal patterns of disease [19].

The periodic emergence of new strains of norovirus may also interact with host immunity in the population to mediate seasonal patterns of disease. Noroviruses are RNA viruses of the family *Caliciviridae* [20], and are classified into one of five genogroups (GI-GV), with human infections principally caused by GI and GII [21]. Genogroup II genotype 4 (GGII.4) viruses predominate and rapidly evolve in response to human population immunity, with new GII.4 variants emerging every two to four years. The emergence of a novel GII.4 virus in 2002/03 was associated with unusual spring/summer seasonality, as well as overall wintertime increase of disease, presumably due to an initial lack of effective population immunity to the emergent variant [22].

The most recent review of global seasonality of norovirus was published over a decade ago [23], before PCR diagnostics were widely available. To update our understanding of the global seasonality of norovirus in the age of modern diagnostics, we conducted a systematic review of the literature and carried out an ecological analysis to identify factors associated with the strength of norovirus seasonality.

## Methods

### Search Strategy and Selection Criteria

We carried out a systematic review of the PubMed Medline database through Endnote X4 software (Carlsbad, CA) in August, 2011 using the search terms “norovirus” and each of the following terms: “ambient temperature,” “climate,” “rain,” “relative humidity,” “season,” and “weather”. Titles and abstracts were screened for relevance by two independent reviewers and original articles were obtained and screened for inclusion according to the following criteria: (i) conducted continuously for one year or more; (ii) monthly data reported on human norovirus counts (cases or outbreaks); and (iii) conducted after 1997 (when modern PCR-based diagnostics for norovirus began widespread use). Non-human and laboratory studies were excluded, as were studies presenting monthly percentages without reporting total numbers of cases or outbreaks. No exclusions were made for language of publication.

Reported monthly norovirus burden measures were extracted using Plot Digitizer (SourceForge.net). If published figures/tables were not available in high enough resolution for digital extraction, we contacted the authors to request their original dataset. If authors did not respond, or if the researchers were unable to provide data in the monthly format, the article was excluded (n = 8). Two datasets (Johansen et al. [24], Hulth et al. [25]) were expanded when authors replied with more inclusive data. If data were reported for several years but aggregated by month, the dataset was included without a specific season-year, and only used in the analysis not requiring monthly data.

### Data Processing

We classified studies by outcome according to whether they reported counts of monthly cases or outbreaks. Case datasets included records of norovirus-positive laboratory samples or individual cases reported through studies of regional/national reporting systems. Outbreak definitions varied among papers, but outbreak data included reports of cases clustered in time and place.

Several data processing steps were carried out to ensure comparability of data. For articles presenting data stratified by genotype/genogroup, counts of cases or outbreaks across all the subgroups were summed for each month. If the study reported results as percentages but included monthly totals, results were converted to monthly counts. Weekly counts were summed over months, and only single instances of redundant datasets (i.e., multiple publications using the same data) were used. If an article reported norovirus burden separately for more than one country, multiple datasets were created, one for each national population.

### Descriptive Analysis: Timing of Seasonal Patterns

We generated three metrics for analysis: (1) *Average Seasonality*: normalized proportion of cases/outbreaks by month summed over all years; (2) *Long-term Seasonal Patterns*: normalized proportion of cases/outbreaks by calendar month of study period; and (3) *Season Strength*: peak to mean ratio of normalized proportion of cases/outbreaks by month. The derivation of these metrics can be found in the Supplemental Material.

### Regression Analysis: Predictors of Seasonal Patterns

For each study location we assembled information on latitude, average winter temperature (°C) (with winter defined as December/January/February and June/July/August for Northern and Southern hemispheres, respectively), peak-to-trough ratio of monthly average temperature (per season year), and average monthly precipitation in the wettest month (cm) using publicly available information for the city in which the study occurred (42% of case and 21% of outbreak datasets) [26], [27]. If three or fewer cities were included in a single study, values were averaged for those cities (one case dataset). If the study included data from greater than three cities or for a region or country as a whole, data for the largest city (by population) was used (53% of case and 69% of outbreak datasets). Weather data were excluded for studies that covered large, nonhomogeneous locations (two cruise ship outbreak studies, one case dataset from China, one case dataset from Brazil, and one outbreak dataset from the USA).

Country-level data included gross-domestic product (GDP in 2011 international dollars) [28], crude birth rate (births/1000 population) [29], and population density (population per square km) [29]. The crude birth rate value was only available in five-year intervals, and was applied accordingly. Population density was the yearly estimate for the first calendar year of a season year (e.g., 1998 estimate used for season year 1998-99).

We also included a binary variable to indicate season-years when new pandemic strains emerged. A *new strain year* was defined as a season-year with an antigenic shift and reports of heightened activity, which occurred in the 2002–2003 and 2006–2007 seasons [22], [30].

We modeled norovirus season strength as a function of the climatic and socio-demographic variables described above. Norovirus season strength was log-transformed in order to normalize its distribution. Separate linear regression models were fitted for cases, outbreaks, and the combined dataset of cases and outbreaks. Bivariate linear analyses were initially performed, with each variable individually predicting the outcome. Multivariate linear models were constructed using forward selection; variables with *p*-values <0·2 in the bivariate analyses were added, with the next most significant variables being added one at a time. Once in the model, variables were not dropped. For the case dataset, no variable had a *p*-value <0·2 in the bivariate models, so we started the multivariate modeling with the most significant variable from the outbreak dataset. We did not include latitude, average winter temperature, and peak to trough temperature ratio in the same model because of collinearity of these three variables.

## Results

**Figure 1:**
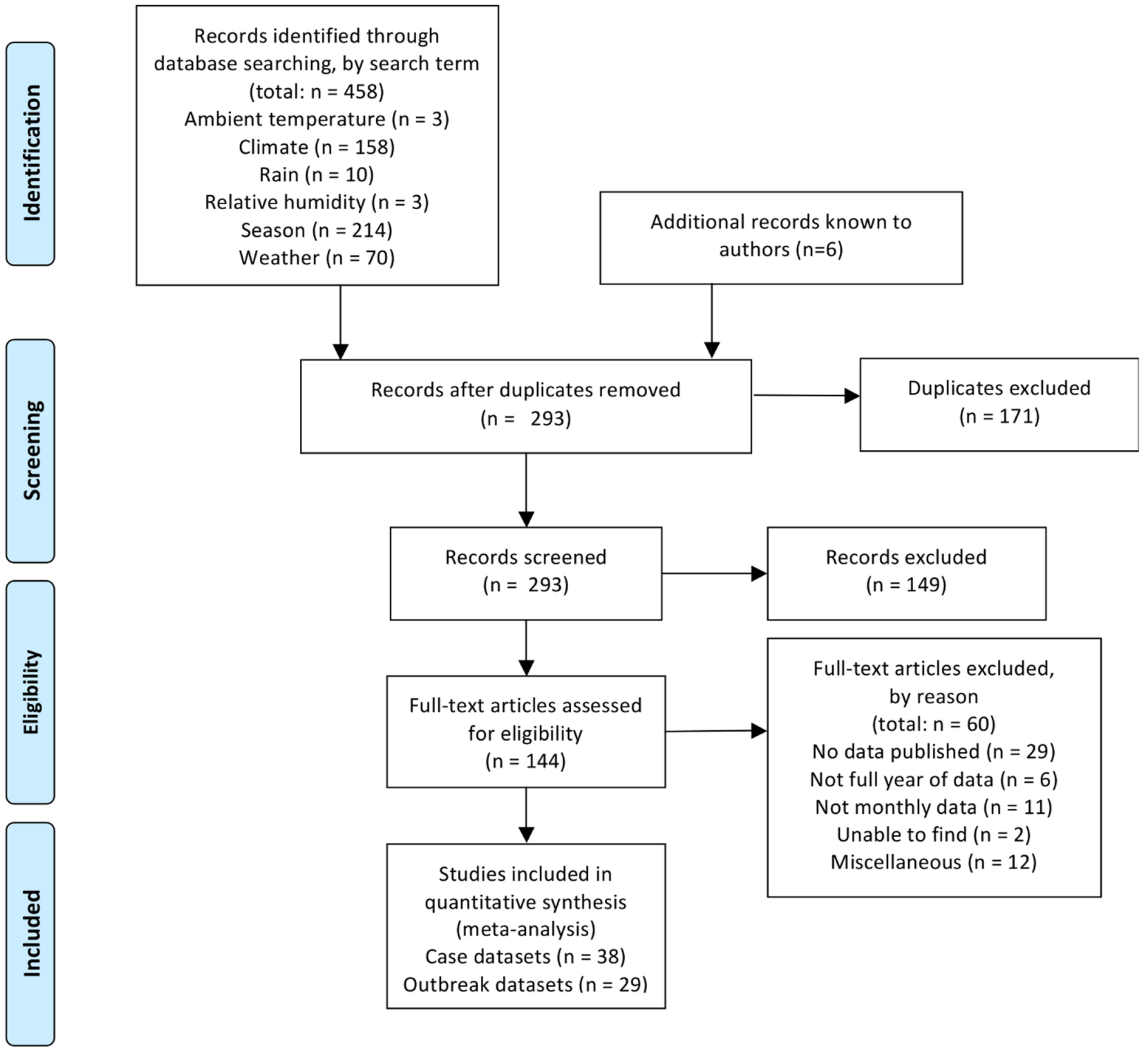
Flow chart of search strategy. Note that final numbers refer to total number of data series included in analyses, whereas other numbers refer to numbers of articles reviewed.

Our original search yielded 458 articles, 171 of which were duplicates, and 149 of which were deemed irrelevant based on review of their title/abstract. Of the remaining 138 articles, 29 did not report data, six did not span an entire year, 11 did not report monthly outcomes, and two could not be located. Fourteen others were excluded for miscellaneous reasons (outcome measures that could not be compared to other studies). Six relevant articles not identified in the search but known to the authors were also included in the review, two datasets were added/removed based on clarifying correspondence with authors, and multiple articles using the same dataset were condensed into a single dataset. We included papers published in all languages including English, Mandarin Chinese, Portuguese, and Polish by translating relevant sections (captions and data presented in tables and/or graphs). The final dataset included 38 case and 29 outbreak series (Figure 1, Table S1).

### Overall Seasonality

**Figure 2:**
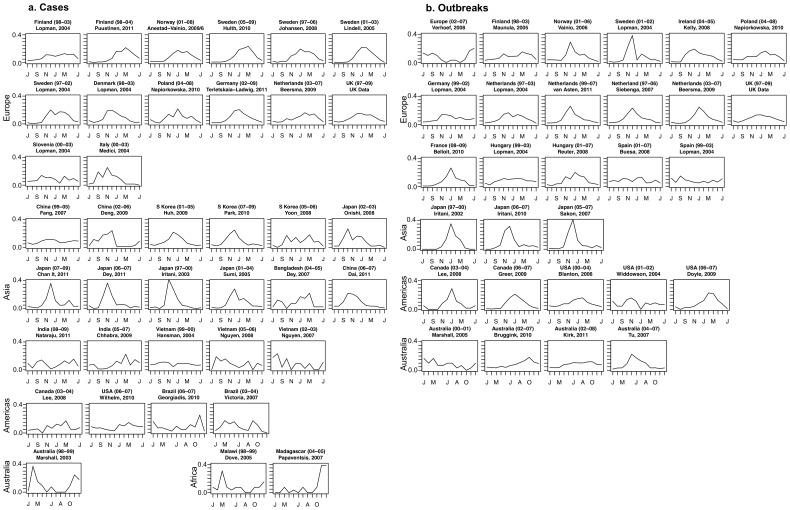
Normalized proportion of a) norovirus cases and b) norovirus outbreaks. Presented by month summed over all years for each study, by location. Within continents, studies are displayed from top to bottom in order of latitude, North to South.

Norovirus cases and outbreaks exhibited a clear seasonality across most of the locations in this study (Figure 2), with a peak in winter months and a trough in summer months. Among the 68 studies analyzed, 52·7% of cases and 41·2% of outbreaks occurred in winter months (December-February in the Northern Hemisphere, June-August in the Southern Hemisphere), and 78·9% of cases and 71·0% of outbreaks occurred in cool months (October-March in the Northern Hemisphere, April-September in the Southern Hemisphere). The average season strength (peak to mean ratio of cases/outbreaks by month) was 3·0 in Europe and 3·6 in Asia; there was not enough data to summarize season strength by region for the Americas, Africa or Australia.

**Figure 3:**
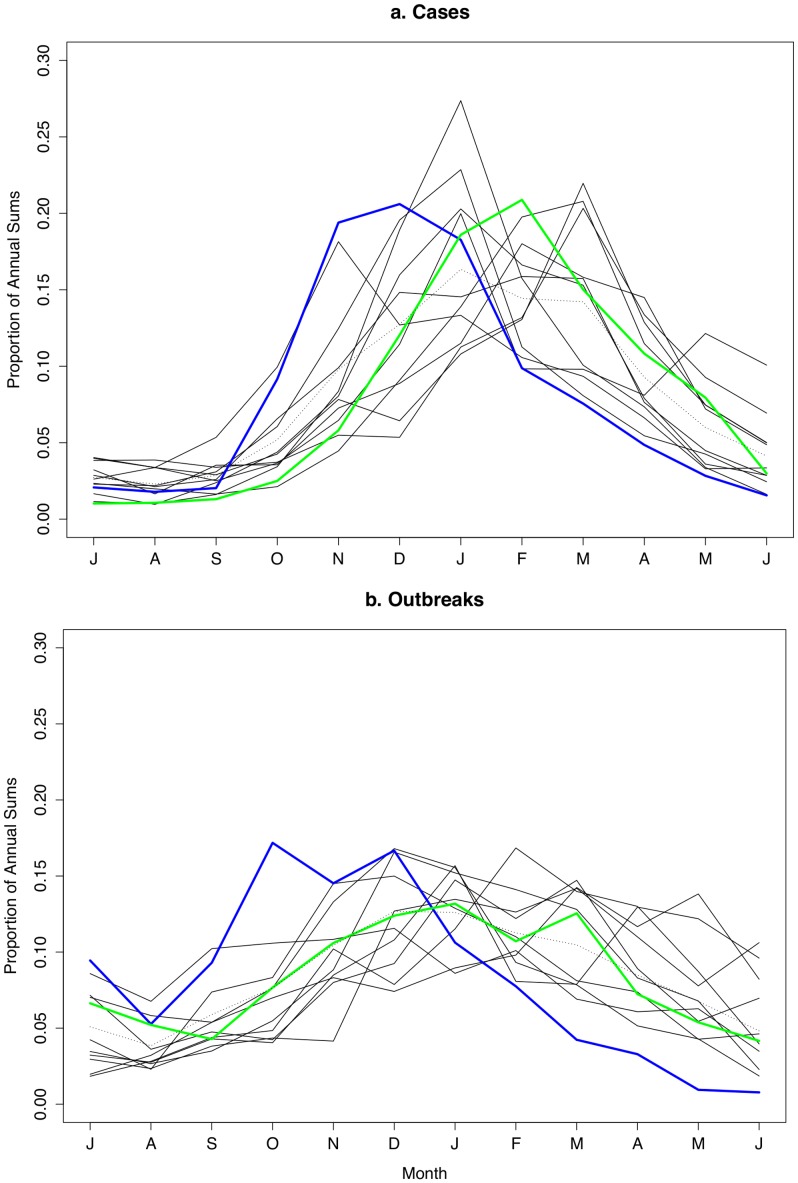
Monthly proportion of a) norovirus cases and b) norovirus outbreaks by season-year in northern hemisphere. New strain years highlighted (Blue = 2002-03; Green = 2006-07). Dotted line indicates studies where monthly datasets were reported as averages over several years.

In the 12 season-year span of northern hemisphere data for cases, seven seasons peaked in December-February (winter), and five peaked in March. For outbreaks, eight seasons peaked in December-February and three peaked in March (Figure 3). There were no studies that reported outbreak data for the 2008–2009 season. Outbreak reports had a somewhat weaker seasonality than case reports (average peak to mean ratio of 2·06 versus 2·35, respectively).

In terms of *new strain years*, both case and outbreak studies showed an earlier seasonality in 2002-03, but not in season-year 2006-07 (Figure 3).

### Determinants of season strength

For outbreaks, norovirus season strength was positively associated with average rainfall in the wettest month, and inversely associated with crude birth rate in both bivariate (rainfall: *R* ^2^ = 0·0564, *p* = 0·0096; crude birth rate: *R* ^2^ = 0·0941, *p* = 0·0011) and multivariate analyses (*R* ^2^ = 0·1848, rainfall: *p* = 0·0005, crude birth rate: *p*<0·0001) (Table 1). None of the covariates examined was associated with season strength from cases studies in either bivariate or multivariate analyses.

**Table 1:** Linear regression model results of associations between potential weather, socio-demographic, and viral predictors with norovirus season strength.

	Bivariate – percent change (95%CI)			Multivariate – percent change (95%CI)		
Dataset	Cases	Outbreaks	Combined	Cases	Outbreaks	Combined
Latitude (degree)	−0.09 (−0.41, 0.24)	0.06 (−0.16, 0.27)	0.02 (−0.15, 0.19)			
Avg winter temp (°C)	−0.20 (−0.97, 0.58)	−0.69 (−2.04, 0.69)	−0.34 (−1.01, 0.34)			−0.97 (−1.77, −0.17)
Peak/ trough temp ratio (°C)	0.21 (−0.21, 0.64)	−0.28 (−0.73, 0.18)	−0.02 (−0.33, 0.29)			
Avg rain in wettest month (cm)	0.16 (−0.51, 0.84)	1.78 (0.44, 3.13)	0.51 (−0.09, 1.11)	0.67 (−0.32, 1.67)	2.61 (1.16, 4.08)	1.01 (0.29, 1.74)
GDP (per $1,000)	0.00 (0.0, 0.0)	0.00 (0.0, 0.0)	0.00 (0.0, 0.0)	0.00 (0.0, 0.0)		
Crude birth rate (births/1,000 ppl)	0.03 (−1.12, 1.19)	−7.03 (−10.93, −2.96)	−0.44 (−1.55, 0.69)		−8.35 (−12.11, −4.43)	
Pop density (pl/km^2^)	0.02 (−0.01, 0.06)	0.01 (−0.03, 0.05)	0.02 (−0.01, 0.05)		−0.04 (−0.08, 0.01)	
New strain year	−7.18 (−19.93, 7.6)	−4.9 (−17.89, 10.15)	−6.04 (−15.23, 4.15)			

Norovirus season strength is defined as peak to mean ratio of normalized monthly proportion of norovirus cases or outbreaks for each season-year. Estimates are beta coefficients and can be interpreted as expected percent change in norovirus season strength for a one-unit increase in the predictor variable. Results for multivariate linear regression models are only for variables included in the final models based on forward selection (see text for further explanation of modeling strategy).

In the combined case and outbreak analysis, no covariates were individually associated with season strength, but in the multivariate model, average rainfall in the wettest month was positively associated (*R* ^2^ = 0·0321, rainfall: *p* = 0·0065, avg winter temp: *p* = 0·0177).

## Discussion

This systematic review and meta-analysis of the seasonality of norovirus highlights a pattern of distinct but variable global seasonality, with a peak in winter months (December-February in the Northern Hemisphere, June-August in the Southern Hemisphere) in most settings. Approximately half of all the cases and outbreaks occurred in winter months while approximately three-quarters occurred in cool months (October-March in the Northern Hemisphere, April-September in the Southern Hemisphere).

Our findings are broadly consistent with the notion that norovirus incidence peaks in the winter, at least in temperate climates[23]. In Europe, where there were more data over a range of years and climates, the relative strength of the norovirus peak was clearly observed during winter months (Figure 2). We did not detect strong, consistent associations between socio-demographic or weather variables with the strength of the norovirus season.

This review substantially expands a previous study on the global seasonality of norovirus by Mounts et al. [23], published over a decade ago. In the intervening years, a relative abundance of surveillance data have become available, largely due to the expanded availability of sensitive diagnostic assays and their widespread use by public health agencies. Mounts et al. reviewed 14 studies, evenly split between case-based and outbreak-based studies. Our study started where the previous review left off, in 1997, and included over four times as many data series. In addition to the large number of studies from Europe, our review included a considerable number of studies from Asia, representing the range of wealth in the region. However, there remains little published data from Africa or South America, or for tropical regions in general. The data that are available from these locations are from highly diverse settings and generally of short duration, limiting our ability to differentiate between the effects of meteorological and demographic characteristics on norovirus seasonality in these areas (see Figure S1).

Based on two papers from Victoria, Australia, it has been posited that norovirus incidence in the southern hemisphere contrasts with the northern hemisphere by peaking in warmer months [10]. In our review, neither these data from Australia nor other southern hemisphere settings (Brazil, Malawi, Madagascar) exhibited a consistent summer or winter peak once normalized across years (Figure 2). It is unclear if this lack of observed seasonality in southern hemisphere settings is a result of different epidemiological patterns, or a lack of robust data.

The majority of studies included in our review were from regional or national surveillance systems, capturing outbreaks from schools, nursing homes, hospitals, and the general community, among other settings. Other studies that depart from the general pattern of wintertime seasonality are those reporting outbreaks on cruise ships. These show an inverted seasonal pattern, which likely reflects the seasonality of cruise voyages in their respective geographic areas (Caribbean, Mediterranean).

Somewhat unexpectedly, outbreak studies exhibited weaker seasonality than case studies. Outbreaks may exhibit different seasonal patterns than cases since population sizes in settings at risk of outbreaks may vary throughout the course of the year (e.g. elevated in summer for cruise ships and in winter for healthcare facilities). In addition, case datasets include active surveillance for sporadic cases (e.g., collecting and testing stool from diarrhea patients in emergency departments) as well as passive laboratory surveillance (all stools sent for testing). The stools sent to laboratories for testing might originate from outbreak investigations. If so, multiple norovirus positive stools reported in these situations could inflate the observed seasonality in studies that we classified as case data series. Regardless, the proportion of illnesses that get recorded may differ between case surveillance and outbreak surveillance, which could lead to differences in estimates of observed seasonal strengths.

The emergence of a new GII.4 variant in 2002 precipitated a break with typical seasonality, presumably due to faster transmission resulting from this virus's escape from population immunity [22]. However, this pattern was not observed in 2006/07, where, if anything, a later-than-usual peak was observed, possibly implying that the new strain emerged later in the season. Thus, we speculate that escaping population immunity may result in a departure from typical seasonality, but the occurrence of this phenomenon might depend on when in the norovirus season the new strain first appears.

It is important to note that we do not propose our predictor variables, including latitude, average winter temperature, and peak-to-trough temperature ratio, as part of the causal mechanism of norovirus gastroenteritis. Rather, we attempted to use them as possible indicators of temperature-mediated transmission. On a broad ecological scale, our results point to a possible association between season strength and both rain in the wettest month and crude birth rate. These results are in agreement with some local studies, including that of Marshall and Bruggink [10], which suggested that rainfall may be a more important predictor of norovirus seasonality than temperature in Australia. Interestingly, lower birth rates have also been associated with stronger seasonality of rotavirus, at least in some settings [14], [19], [31]. Birth rates, which were inversely associated with norovirus in some of our models, are thought to influence seasonality via replenishment of susceptible individuals in the population. However, in general, caution should be taken in interpreting our results: most of the variability observed in the data was not explained by variables included in our models.

It is also important to note that our study did not directly assess factors that may lead to higher numbers of norovirus cases. Instead, we examined meteorological characteristics of a location and demographic characteristics of that location's population that could potentially influence transmission rates and in turn, the degree of the seasonal variation in human illness. Weather and demographic characteristics may or may not have a directly causal relationship with norovirus transmissibility, but even if they do, it can be exceedingly difficult to detect such an association since epidemiological data represent case/outbreak events, not transmission events *per se* [32]. Moreover, we used monthly averages of meteorological characteristics as potential predictors of seasonality, instead of records of the actual meteorological conditions matched in time, which is an oversimplification of the actual climatic conditions at the time of the case/outbreak. Country-level demographic descriptors may also be too broad to capture the influence of these factors at the local level. Ideally, both epidemiological (outcome) and weather/demographic (exposure) would be specific to areas that are large enough to capture general disease patterns but small enough to have homogenous meteorological and populations characteristics, and would also be matched in time.

In order to better understand the global seasonality of norovirus disease and the factors influencing that seasonality, there is a need for additional studies in developing and southern hemisphere countries. There was limited variability in the data from our review, since the majority of data were from Europe and North America - temperate regions with similar levels of income and health/sanitation infrastructure. Regardless, this review makes it clear that norovirus is a wintertime phenomenon, at least in the temperate northern hemisphere. Understanding the seasonality of norovirus offers insight into the factors that promote its transmission.

## Supporting Information

Figure S1 **Scatterplots of a) latitude, b) average winter temperature, c) summer to winter temperature ratio, d) peak monthly rainfall, e) crude birth rate, f) population density, and g) GDP against norovirus season strength with regression line.**
(TIF)

Table S1 **Summary of Studies Included in Review.**
(DOC)
