# Supplementary figures and images for: A Systematic Review and Meta-Analysis of the Global Seasonality of Norovirus
Source: PLoS One. 2013 Oct 2;8(10):e75922. doi: 10.1371/journal.pone.0075922 (PMC3788804; doi:10.1371/journal.pone.0075922)

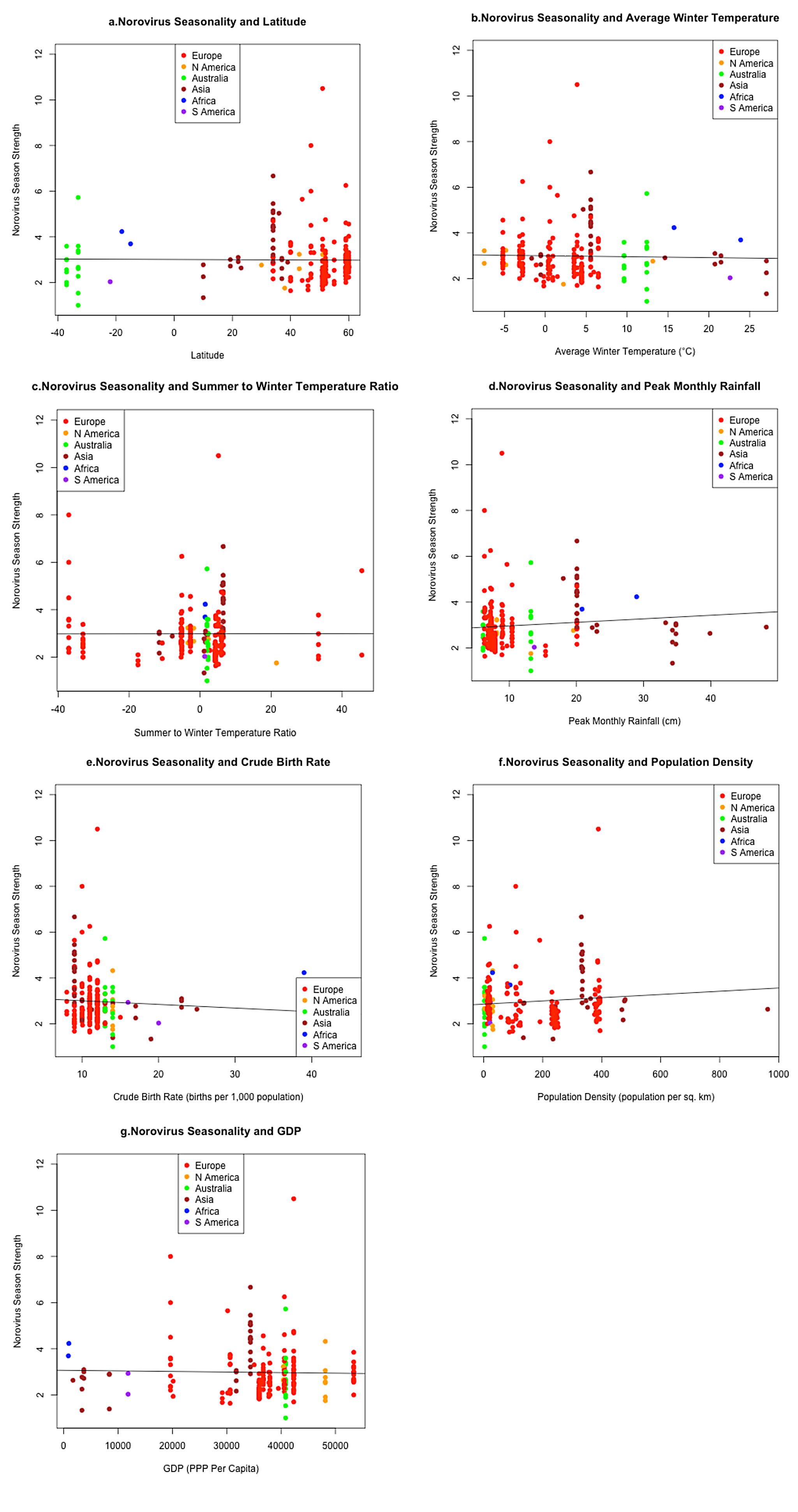

Supplement: Figure S1 — Scatterplots of a) latitude, b) average winter temperature, c) summer to winter temperature ratio, d) peak monthly rainfall, e) crude birth rate, f) population density, and g) GDP against norovirus season strength with regression line. (TIF) [file pone.0075922.s001.tif]
