# Supplementary material for: A Systematic Review and Meta-Analysis of the Global Seasonality of Norovirus
Source: PLoS One. 2013 Oct 2;8(10):e75922. doi: 10.1371/journal.pone.0075922 (PMC3788804; doi:10.1371/journal.pone.0075922)
Supplement: Table S1 — Summary of Studies Included in Review. (DOC) [file pone.0075922.s002.doc]

| **TECHNICAL APPENDIX AND OTHER MATERIALS**  **Table S1: Summary of Studies Included in Review** | | | | | | |  |  |  |  |  |  |  |  |  |  |
| --- | --- | --- | --- | --- | --- | --- | --- | --- | --- | --- | --- | --- | --- | --- | --- | --- |
| **Author** | **Year_Pub** | **Ref** | **Continent** | **Country** | **City** | **Season-Years** | **Outcome** | **Latitude (degree)** | **Avg. Winter Temp (°C)** | **Summer/**  **Winter Temp (°C)** | **Avg rain in wettest month (cm)** | **New Strain Year (Y/N)** | **Crude Birth Rate (births per 1,000 pop.)** | **Pop. Density (pop. per sq. km.)** | **GDP**  **(PPP per capita)** | **# Cases/**  **Outbreaks** |
| Anestad-Vainio |  | , | Europe | Norway | NA | 01-08 | Cases | 59 | -3 | -5 | 9 | N | 12.8 | 11.9 | 53376 | 5274 |
| Beersma | 2009 |  | Europe | Netherlands | Rotterdam | 03-07 cases, 03-07 outbreaks | Cases,  Outbreaks | 51 | 4 | 5 | 8.9 | N | 12.1 | 388 | 42331 | 224 cases, 559 outbreaks |
| Belliot | 2010 |  | Europe | France | NA | 08-09 | Outbreaks | 48 | 4 | 5 | 6.5 | N | 12.8 | 109.2 | 35049 | 238 |
| Blanton | 2006 |  | N America | USA | NA | 00-04 | Outbreaks | NA | NA | NA | NA | N | 14.1 | 30.2 | 48147 | 180 |
| Bruggink | 2010 |  | Australia | Australia | Victoria | 02-07 | Outbreaks | -37 | 10 | 2 | 6 | Y | 13.3 | 2.6 | 40836 | 767 |
| Buesa | 2008 |  | Europe | Spain | Catalonia, Valencia | 01-07 | Outbreaks | 40 | 6 | 4 | 6.4 | N | 10.2 | 83.5 | 30622 | 194 |
| Chan_It | 2011 |  | Asia | Japan | Tokyo, Sapporo, Saga, Osaka, Maizuru | 07-09 | Cases | 34 | 6 | 7 | 20.1 | N | 9 | 333.6 | 34362 | 254 |
| Chhabra | 2009 |  | Asia | India | Pune, Nagpur, Aurangabad | 05-07 | Cases | 19.3 | 21 | 2 | 23 | N | 25 | 336.2 | 3703 | 89 |
| Dai | 2011 |  | Asia | China | Jiangmen City | 06-07 | Cases | 22 | 15 | 2 | 48.3 | N | 14 | 134.5 | 8394 | 115 |
| Deng | 2009 |  | Asia | China | Beijing | Avg (02-06) | Cases | 39 | -2 | -8 | 22.4 | NA | 14 | 134.5 | 8394 | 79 |
| Dey | 2007 |  | Asia | Bangladesh | Dhaka | 04-05 | Cases | 23 | 21 | 2 | 39.9 | N | 25.3 | 942.7 | 1697 | 41 |
| Dey | 2011 |  | Asia | Japan | Maizuru, Tokyo, Sapporo, Saga, Osaka | 06-07 | Cases | 34 | 6 | 7 | 20.1 | Y | 9 | 333.6 | 34362 | 98 |
| Dove | 2005 |  | Africa | Malawi | Blantyre | 98-99 | Cases | -15 | 24 | 1 | 21 | N | 45 | 89.6 | 853 | 26 |
| Doyle | 2009 |  | N America | USA | Florida | 06-07 | Outbreaks | 30 | 13 | 2 | 19.6 | Y | 14.1 | 30.2 | 48147 | 113 |
| Fang | 2007 |  | Asia | China | 13 regions listed | Avg (99-05) | Cases | NA | NA | NA | NA | NA | 14 | 134.5 | 8394 | 777 |
| Georgiadis | 2010 |  | S America | Brazil | NA | 06-07 | Cases | NA | NA | NA | NA | N | 19.2 | 21.3 | 11846 | 48 |
| Greer | 2009 |  | N America | Canada | Toronto | 06-08 | Outbreaks | 43 | -5 | -3 | 8.1 | Y | 11.1 | 3.2 | 40458 | 247 |
| Hansman | 2004 |  | Asia | Vietnam | Ho Chi Minh | 99-00 | Cases | 10 | 27 | 1 | 34.3 | N | 17.8 | 245.5 | 3355 | 1368 |
| Huh | 2009 |  | Asia | S Korea | Gyeonggi province | Avg (01-05) | Cases | 37 | -1 | -12 | 34.8 | NA | 11.3 | 468.4 | 31754 | 367 |
| Hulth | 2010 |  | Europe | Sweden | NA | 05-09 | Cases | 59 | -3 | -5 | 7.2 | N | 11 | 20 | 40614 | 22895 |
| Iritani | 2002 |  | Asia | Japan | Osaka City | 97-00 | Outbreaks | 34 | 6 | 7 | 20.1 | N | 9 | 333.6 | 34362 | 62 |
| Iritani | 2003 |  | Asia | Japan | Osaka City | 97-00 | Cases | 34 | 6 | 7 | 20.1 | N | 9 | 333.6 | 34362 | 93 |
| Iritani | 2010 |  | Asia | Japan | Osaka City | 06-07 | Outbreaks | 34 | 6 | 7 | 20.1 | Y | 9 | 333.6 | 34362 | 144 |
| Johansen | 2008 |  | Europe | Sweden | NA | 97-06 | Cases | 59 | -3 | -5 | 7.2 | N | 11 | 20 | 40614 | 4081 |
| Kelly | 2008 |  | Europe | Ireland | NA | 04-05 | Outbreaks | 53 | 6 | 3 | 7.6 | N | 15.4 | 57.2 | 39508 | 263 |
| Kirk | 2010 |  | Australia | Australia | Long-term care facilities | 02-08 | Outbreaks | -33 | 12 | 2 | 13.2 | Y | 13.3 | 2.6 | 40836 | 1147 |
| Lee | 2008 |  | N America | Canada | Alberta | 03-04 cases, 03-04 outbreaks | Cases, Outbreaks | 51 | -7 | -2 | 8.1 | N | 11.1 | 3.2 | 40458 | 141 cases, 78 outbreaks |
| Lindell | 2005 |  | Europe | Sweden | mostly Stockholm | 01-03 | Cases | 59 | -3 | -5 | 7.2 | N | 11 | 20 | 40614 | 878 |
| Lopman | 2004 |  | Europe | Denmark | NA | 98-03 | Cases | 55 | 1 | 33 | 7.4 | N | 12.1 | 125.1 | 37742 | 1896 |
| Lopman | 2004 |  | Europe | Finland | NA | 98-03 | Cases | 60 | -5 | -3 | 7.4 | N | 11.2 | 15.4 | 36723 | 1629 |
| Lopman | 2004 |  | Europe | Germany | NA | 99-02 | Outbreaks | 52 | 0 | -33 | 6.9 | N | 8.9 | 230.9 | 37936 | 376 |
| Lopman | 2004 |  | Europe | Hungary | NA | 99-03 | Outbreaks | 47 | 1 | -37 | 6.3 | N | 9.7 | 109 | 19647 | 184 |
| Lopman | 2004 |  | Europe | Netherlands | NA | 97-03 | Outbreaks | 52 | 4 | 6 | 10.4 | N | 12.1 | 388 | 42331 | 270 |
| Lopman | 2004 |  | Europe | Slovenia | NA | 00-03 | Cases, Outbreaks | 46 | 0 | -18 | 15.4 | N | 9.3 | 98.6 | 29179 | 808 |
| Lopman | 2004 |  | Europe | Spain | NA | 99-03 | Outbreaks | 40 | 6 | 4 | 6.4 | N | 10.2 | 83.5 | 30622 | 245 |
| Lopman | 2004 |  | Europe | Sweden | NA | 97-02cases, 01-02 outbreaks | Cases,  Outbreaks | 59 | -3 | -5 | 7.2 | Y | 11 | 20 | 40614 | 3213 cases, 1192 outbreaks |
| Marshall | 2003 |  | Australia | Australia | Melbourne | 98-99 | Cases | -37 | 10 | 2 | 6 | N | 13.3 | 2.6 | 40836 | 79 |
| Marshall | 2005 |  | Australia | Australia | Victoria | 00-01 | Outbreaks | -37 | 10 | 2 | 6 | N | 13.3 | 2.6 | 40836 | 30 |
| Maunula | 2005 |  | Europe | Finland | Helsinki | 98-03 | Outbreaks | 60 | -5 | -3 | 7.4 | N | 11.2 | 15.4 | 36723 | 252 |
| Medici | 2004 |  | Europe | Italy | Parma | 00-03 | Cases | 44 | 1 | 46 | 9.7 | N | 9.3 | 193 | 30166 | 63 |
| Napiow… | 2010 |  | Europe | Poland | NA | Avg (04-08) | Cases, Outbreaks | 52 | -1 | -11 | 7.6 | NA | 10.1 | 118.3 | 20137 | 2724 cases, 130 outbreaks |
| Nataraju | 2011 |  | Asia | India | Kolkata | 08-09 | Cases | 22 | 21 | 2 | 33.3 | N | 25 | 336.2 | 3703 | 78 |
| Nguyen | 2007 |  | Asia | Vietnam | Ho Chi Minh | 02-03 | Cases | 10 | 27 | 1 | 34.3 | N | 17.8 | 245.5 | 3355 | 1402 |
| Nguyen | 2008 |  | Asia | Vietnam | Ho Chi Minh | 05-06 | Cases | 10 | 27 | 1 | 34.3 | Y | 17.8 | 245.5 | 3355 | 32 |
| Onishi | 2008 |  | Asia | Japan | Soma | 02-03 | Cases | 36 | 5 | 6 | 18 | Y | 9 | 333.6 | 34362 | 105 |
| Papaventsis | 2007 |  | Africa | Madagascar | Antananarivo | 04-05 | Cases | -18 | 16 | 1 | 29 | N | 39.3 | 28.8 | 943 | 14 |
| Park | 2010 |  | Asia | S Korea | 5 hosp in 3 areas (Seoul, Gyenogsi-do state, Gwangwon-do state) | 07-09 | Cases | 37 | -1 | -12 | 34.8 | N | 11.3 | 468.4 | 31754 | 1169 |
| Puustinen | 2011 |  | Europe | Finland | NA | 98-04 | Cases | 60 | -5 | -3 | 7.4 | N | 11.2 | 15.4 | 36723 | 765 |
| Reuter | 2008 |  | Europe | Hungary | NA | 01-07 | Outbreaks | 47 | 1 | -37 | 6.3 | N | 9.7 | 109 | 19647 | 301 |
| Sakon | 2007 |  | Asia | Japan | Osaka City | 05-07 | Outbreaks | 34 | 6 | 7 | 20.1 | N | 9 | 333.6 | 34362 | 525 |
| Siebenga | 2007 |  | Europe | Netherlands | NA | 97-06 | Outbreaks | 34 | 6 | 7 | 20.1 | N | 12.1 | 388 | 42331 | 605 |
| Sumi | 2005 |  | Asia | Japan | NA | 01-04 | Cases | 34 | 6 | 7 | 20.1 | N | 9 | 333.6 | 34362 | 3413 |
| Terletskaia-Ladwig | 2011 |  | Europe | Germany | Baden-Württemberg, | 02-09 | Cases | 52 | 0 | -33 | 6.9 | Y | 8.9 | 230.9 | 37936 | 682562 |
| Tu | 2007 |  | Australia | Australia | New South Wales | 04-07 | Outbreaks | -33 | 12 | 2 | 13 | Y | 13.3 | 2.6 | 40836 | 734 |
| UK Data[[1]](#footnote-2) | 2003/  2009 | , | Europe | England/ Wales | NA | 97-09 cases, 97-09 outbreaks | Cases,  Outbreaks | 51 | 4 | 4 | 7.9 | N | 12 | 246 | 35974 | 47109 cases, 4148 outbreaks |
| Vainio | 2006 |  | Europe | Norway | NA | 01-06 | Outbreaks | 59 | -3 | -5 | 9 | N | 12.8 | 11.9 | 53376 | 197 |
| vanAsten | 2011 |  | Europe | Netherlands | NA | 99-07 | Outbreaks | 52 | 4 | 6 | 10.4 | N | 12.1 | 388 | 42331 | 746 |
| Verhoef | 2008 |  | Europe | NA | NA | 02-07 | Outbreaks | NA | NA | NA | NA | Y | NA | NA | NA | 29 |
| Victoria | 2007 |  | S America | Brazil | Rio de Janeiro | 03-04 | Cases | -22 | 23 | 1 | 13.7 | N | 19.2 | 21.3 | 11846 | 65 |
| Widdowson | 2004 |  | N America | USA | cruiseships | 01-02 | Outbreaks | NA | NA | NA | NA | Y | 14.1 | 30.2 | 48147 | 44 |
| Wilhelm | 2010 |  | N America | USA | Charleston | 06-07 | Cases | 38 | 2 | 22 | 13.2 | N | 14.1 | 30.2 | 48147 | 979 |
| Yoon | 2008 |  | Asia | S Korea | NA | 05-06 | Cases | 37 | -1 | -12 | 34.8 | Y | 11.3 | 468.4 | 31754 | 114 |

**CALCULATIONS**

Season strength is defined as peak to mean normalized monthly cases/outbreaks. We normalized monthly count data as the proportion of cases or outbreaks that occur in a given month such that the sum for the entire study period of that article equals 1. Normalized case/outbreak counts serves as an indicator of norovirus activity in a given month and may vary across years to account for annual variation within a given study. We used the following equation to normalize monthly counts of cases/outbreaks (*P)*, accounting for the duration of the study:

$$P_{ym}=\frac{C_{ym}}{\sum C}\frac{N}{12}$$

were *P_ym_* is the normalized monthly counts in year *y* and month *m*, where *C_ym_* is the number of cases/outbreaks in year-month *ym* and *N* is the total number of months in the study.

Overall seasonality: We normalized the proportion of cases or outbreaks occurring in a given calendar month (
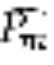
) for multi-year studies as follows:

$$P_{m}=\frac{\sum_{y} P_{ym}}{N_{m}}$$

Averages of normalized data were calculated, rather than normalizing averages, because not all studies had multiple years of data. This statistic allowed us to compare the norovirus seasonality in a typical year for each study location.

Long-term seasonal patterns: In order to compare the seasonality of norovirus across different years, we calculated a weighted normalized summation of cases or outbreaks, calculated as follows:

$$PW_{m}=\sum_{x} P{}_{ymx}\frac{cases_{x}}{\sum_{x} cases}$$

Here *PW_m_* presents a single value for each month in the study period based on the normalized monthly counts for all studies *x* weighted on the relative size of each study.

**SUPPLEMENTAL REFERENCES**

S1. Anestad G, Vainio K, Hungnes O (2009) Interference between outbreaks of epidemic viruses: additional Norwegian observations. Scand J Infect Dis 41: 381-382.

S2. Vainio K, Myrmel M (2006) Molecular epidemiology of norovirus outbreaks in Norway during 2000 to 2005 and comparison of four norovirus real-time reverse transcriptase PCR assays. J Clin Microbiol 44: 3695-3702.

S3. Beersma MF, Schutten M, Vennema H, Hartwig NG, Mes TH, et al. (2009) Norovirus in a Dutch tertiary care hospital (2002-2007): frequent nosocomial transmission and dominance of GIIb strains in young children. J Hosp Infect 71: 199-205.

S4. Belliot G, Kamel AH, Estienney M, Ambert-Balay K, Pothier P (2010) Evidence of emergence of new GGII.4 norovirus variants from gastroenteritis outbreak survey in France during the 2007-to-2008 and 2008-to-2009 winter seasons. J Clin Microbiol 48: 994-998.

S5. Blanton LH, Adams SM, Beard RS, Wei G, Bulens SN, et al. (2006) Molecular and epidemiologic trends of caliciviruses associated with outbreaks of acute gastroenteritis in the United States, 2000-2004. J Infect Dis 193: 413-421.

S6. Bruggink LD, Marshall JA (2010) The incidence of norovirus-associated gastroenteritis outbreaks in Victoria, Australia (2002-2007) and their relationship with rainfall. Int J Environ Res Public Health 7: 2822-2827.

S7. Buesa J, Montava R, Abu-Mallouh R, Fos M, Ribes JM, et al. (2008) Sequential evolution of genotype GII.4 norovirus variants causing gastroenteritis outbreaks from 2001 to 2006 in Eastern Spain. J Med Virol 80: 1288-1295.

S8. Chan-It W, Thongprachum A, Okitsu S, Nishimura S, Kikuta H, et al. (2011) Detection and genetic characterization of norovirus infections in children with acute gastroenteritis in Japan, 2007-2009. Clin Lab 57: 213-220.

S9. Chhabra P, Dhongade RK, Kalrao VR, Bavdekar AR, Chitambar SD (2009) Epidemiological, clinical, and molecular features of norovirus infections in western India. J Med Virol 81: 922-932.

S10. Dai YC, Hu GF, Zhang XF, Song CL, Xiang WL, et al. (2011) Molecular epidemiology of norovirus gastroenteritis in children in Jiangmen, China, 2005-2007. Arch Virol.

S11. Deng L, Jia LY, Qian Y, Chen DM, Zhang Y, et al. (2009) [Comparative analysis on clinical manifestations for gastroenteritis caused by norovirus and rotavirus]. Zhonghua Liu Xing Bing Xue Za Zhi 30: 398-401.

S12. Dey SK, Nguyen TA, Phan TG, Nishio O, Salim AF, et al. (2007) Molecular and epidemiological trend of norovirus associated gastroenteritis in Dhaka City, Bangladesh. J Clin Virol 40: 218-223.

S13. Dey SK, Phan TG, Mizuguchi M, Okitsu S, Ushijima H (2011) Genetic diversity and emergence of norovirus GII/4-2006b in Japan during 2006-2007. Clin Lab 57: 193-199.

S14. Dove W, Cunliffe NA, Gondwe JS, Broadhead RL, Molyneux ME, et al. (2005) Detection and characterization of human caliciviruses in hospitalized children with acute gastroenteritis in Blantyre, Malawi. J Med Virol 77: 522-527.

S15. Doyle TJ, Stark L, Hammond R, Hopkins RS (2009) Outbreaks of noroviral gastroenteritis in Florida, 2006-2007. Epidemiol Infect 137: 617-625.

S16. Fang ZY, Xie HP, Lv HX, Zhang Q, Duan ZJ, et al. (2007) [Investigation of human calicivirus (HuCV) diarrhea among infantile and young children in China, 1999--2005]. Bing Du Xue Bao 23: 9-15.

S17. Georgiadis S, Pilger DA, Pereira F, Cantarelli VV (2010) [Molecular evaluation of norovirus in patients with acute gastroenteritis]. Rev Soc Bras Med Trop 43: 277-280.

S18. Greer AL, Drews SJ, Fisman DN (2009) Why "winter" vomiting disease? Seasonality, hydrology, and Norovirus epidemiology in Toronto, Canada. Ecohealth 6: 192-199.

S19. Hansman GS, Doan LT, Kguyen TA, Okitsu S, Katayama K, et al. (2004) Detection of norovirus and sapovirus infection among children with gastroenteritis in Ho Chi Minh City, Vietnam. Arch Virol 149: 1673-1688.

S20. Huh JW, Kim WH, Moon SG, Lee JB, Lim YH (2009) Viral etiology and incidence associated with acute gastroenteritis in a 5-year survey in Gyeonggi province, South Korea. J Clin Virol 44: 152-156.

S21. Hulth A, Andersson Y, Hedlund KO, Andersson M (2010) Eye-opening approach to norovirus surveillance. Emerg Infect Dis 16: 1319-1321.

S22. Iritani N, Seto Y, Kubo H, Haruki K, Ayata M, et al. (2002) Prevalence of "Norwalk-like virus" infections in outbreaks of acute nonbacterial gastroenteritis observed during the 1999-2000 season in Osaka City, Japan. J Med Virol 66: 131-138.

S23. Iritani N, Seto Y, Kubo H, Murakami T, Haruki K, et al. (2003) Prevalence of Norwalk-like virus infections in cases of viral gastroenteritis among children in Osaka City, Japan. J Clin Microbiol 41: 1756-1759.

S24. Iritani N, Kaida A, Kubo H, Abe N, Goto K, et al. (2010) Molecular epidemiology of noroviruses detected in seasonal outbreaks of acute nonbacterial gastroenteritis in Osaka City, Japan, from 1996-1997 to 2008-2009. J Med Virol 82: 2097-2105.

S25. Johansen K, Mannerqvist K, Allard A, Andersson Y, Burman LG, et al. (2008) Norovirus strains belonging to the GII.4 genotype dominate as a cause of nosocomial outbreaks of viral gastroenteritis in Sweden 1997--2005. Arrival of new variants is associated with large nation-wide epidemics. J Clin Virol 42: 129-134.

S26. Kelly S, Foley B, Dunford L, Coughlan S, Tuite G, et al. (2008) Establishment of a national database to link epidemiological and molecular data from norovirus outbreaks in Ireland. Epidemiol Infect 136: 1472-1479.

S27. Kirk MD, Fullerton KE, Hall GV, Gregory J, Stafford R, et al. (2010) Surveillance for outbreaks of gastroenteritis in long-term care facilities, Australia, 2002-2008. Clin Infect Dis 51: 907-914.

S28. Lee BE, Preiksaitis JK, Chui N, Chui L, Pang XL (2008) Genetic relatedness of noroviruses identified in sporadic gastroenteritis in children and gastroenteritis outbreaks in northern Alberta. J Med Virol 80: 330-337.

S29. Lindell AT, Grillner L, Svensson L, Wirgart BZ (2005) Molecular epidemiology of norovirus infections in Stockholm, Sweden, during the years 2000 to 2003: association of the GGIIb genetic cluster with infection in children. J Clin Microbiol 43: 1086-1092.

S30. Lopman B, Vennema H, Kohli E, Pothier P, Sanchez A, et al. (2004) Increase in viral gastroenteritis outbreaks in Europe and epidemic spread of new norovirus variant. Lancet 363: 682-688.

S31. Marshall JA, Hellard ME, Sinclair MI, Fairley CK, Cox BJ, et al. (2003) Incidence and characteristics of endemic Norwalk-like virus-associated gastroenteritis. J Med Virol 69: 568-578.

S32. Marshall JA, Dimitriadis A, Wright PJ (2005) Molecular and epidemiological features of norovirus-associated gastroenteritis outbreaks in Victoria, Australia in 2001. J Med Virol 75: 321-331.

S33. Maunula L, Von Bonsdorff CH (2005) Norovirus genotypes causing gastroenteritis outbreaks in Finland 1998-2002. J Clin Virol 34: 186-194.

S34. Medici MC, Martinelli M, Arcangeletti MC, Pinardi F, De Conto F, et al. (2004) Epidemiological aspects of human rotavirus infection in children hospitalized with acute gastroenteritis in an area of northern Italy. Acta Biomed 75: 100-106.

S35. Napiorkowska A, Sadkowska-Todys M (2010) [Epidemiological situation of human norovirus infections in Poland during 2004-2008]. Przegl Epidemiol 64: 27-33.

S36. Nataraju SM, Pativada M, Chatterjee D, Nayak MK, Ganesh B, et al. (2011) Molecular epidemiology of norovirus infections in children and adults: sequence analysis of region C indicates genetic diversity of NVGII strains in Kolkata, India. Epidemiol Infect 139: 910-918.

S37. Nguyen TA, Yagyu F, Okame M, Phan TG, Trinh QD, et al. (2007) Diversity of viruses associated with acute gastroenteritis in children hospitalized with diarrhea in Ho Chi Minh City, Vietnam. J Med Virol 79: 582-590.

S38. Nguyen TA, Hoang L, Pham le D, Hoang KT, Okitsu S, et al. (2008) Norovirus and sapovirus infections among children with acute gastroenteritis in Ho Chi Minh City during 2005-2006. J Trop Pediatr 54: 102-113.

S39. Onishi N, Hosoya M, Matsumoto A, Imamura T, Katayose M, et al. (2008) Molecular epidemiology of norovirus gastroenteritis in Soma, Japan, 2001-2003. Pediatr Int 50: 65-69.

S40. Papaventsis DC, Dove W, Cunliffe NA, Nakagomi O, Combe P, et al. (2007) Norovirus infection in children with acute gastroenteritis, Madagascar, 2004-2005. Emerg Infect Dis 13: 908-911.

S41. Park DJ, Kim JS, Park JY, Kim HS, Song W, et al. (2010) [Epidemiological Analysis of Norovirus Infection between March 2007 and February 2010.]. Korean J Lab Med 30: 647-653.

S42. Puustinen L, Blazevic V, Huhti L, Szakal ED, Halkosalo A, et al. (2011) Norovirus genotypes in endemic acute gastroenteritis of infants and children in Finland between 1994 and 2007. Epidemiol Infect: 1-8.

S43. Reuter G, Pankovics P, Szucs G (2008) Genetic drift of norovirus genotype GII-4 in seven consecutive epidemic seasons in Hungary. J Clin Virol 42: 135-140.

S44. Sakon N, Yamazaki K, Yoda T, Tsukamoto T, Kase T, et al. (2007) Norovirus storm in Osaka, Japan, last winter (2006/2007). Jpn J Infect Dis 60: 409-410.

S45. Siebenga JJ, Vennema H, Duizer E, Koopmans MP (2007) Gastroenteritis caused by norovirus GGII.4, The Netherlands, 1994-2005. Emerg Infect Dis 13: 144-146.

S46. Sumi A, Kobayashi N, Ohtomo N (2005) Proportion of sporadic gastroenteritis cases caused by rotavirus, norovirus, adenovirus and bacteria in Japan from January 2000 to December 2003. Microbiol Immunol 49: 745-756.

S47. Terletskaia-Ladwig E, Eggers M, Enders M, Regnath T (2011) [Epidemiological aspects of gastrointestinal infections.]. Dtsch Med Wochenschr 136: 69-75.

S48. Tu ET, Nguyen T, Lee P, Bull RA, Musto J, et al. (2007) Norovirus GII.4 strains and outbreaks, Australia. Emerg Infect Dis 13: 1128-1130.

S49. Lopman BA, Adak GK, Reacher MH, Brown DW (2003) Two epidemiologic patterns of norovirus outbreaks: surveillance in England and wales, 1992-2000. Emerg Infect Dis 9: 71-77.

S50. Lopman B, Armstrong B, Atchison C, Gray JJ (2009) Host, weather and virological factors drive norovirus epidemiology: time-series analysis of laboratory surveillance data in England and Wales. PLoS One 4: e6671.

S51. van Asten L, Siebenga J, van den Wijngaard C, Verheij R, van Vliet H, et al. (2011) Unspecified gastroenteritis illness and deaths in the elderly associated with norovirus epidemics. Epidemiology 22: 336-343.

S52. Verhoef L, Depoortere E, Boxman I, Duizer E, van Duynhoven Y, et al. (2008) Emergence of new norovirus variants on spring cruise ships and prediction of winter epidemics. Emerg Infect Dis 14: 238-243.

S53. Victoria M, Carvalho-Costa FA, Heinemann MB, Leite JP, Miagostovich M (2007) Prevalence and molecular epidemiology of noroviruses in hospitalized children with acute gastroenteritis in Rio de Janeiro, Brazil, 2004. Pediatr Infect Dis J 26: 602-606.

S54. Widdowson MA, Cramer EH, Hadley L, Bresee JS, Beard RS, et al. (2004) Outbreaks of acute gastroenteritis on cruise ships and on land: identification of a predominant circulating strain of norovirus--United States, 2002. J Infect Dis 190: 27-36.

S55. Wilhelm CM, Hanna SL, Welch CA, Shahid H, Minnich LL, et al. (2010) Viral gastroenteritis in Charleston, West Virginia, in 2007: from birth to 99 years of age. Infect Control Hosp Epidemiol 31: 816-821.

S56. Yoon JS, Lee SG, Hong SK, Lee SA, Jheong WH, et al. (2008) Molecular epidemiology of norovirus infections in children with acute gastroenteritis in South Korea in November 2005 through November 2006. J Clin Microbiol 46: 1474-1477.

| **Section/topic** | **#** | **Checklist item** | **Reported on page #** |
| --- | --- | --- | --- |
| **TITLE** | | |  |
| Title | 1 | Identify the report as a systematic review, meta-analysis, or both. | 1 |
| **ABSTRACT** | | |  |
| Structured summary | 2 | Provide a structured summary including, as applicable: background; objectives; data sources; study eligibility criteria, participants, and interventions; study appraisal and synthesis methods; results; limitations; conclusions and implications of key findings; systematic review registration number. | 2 |
| **INTRODUCTION** | | |  |
| Rationale | 3 | Describe the rationale for the review in the context of what is already known. | 4 |
| Objectives | 4 | Provide an explicit statement of questions being addressed with reference to participants, interventions, comparisons, outcomes, and study design (PICOS). | 4 |
| **METHODS** | | |  |
| Protocol and registration | 5 | Indicate if a review protocol exists, if and where it can be accessed (e.g., Web address), and, if available, provide registration information including registration number. | N/A |
| Eligibility criteria | 6 | Specify study characteristics (e.g., PICOS, length of follow-up) and report characteristics (e.g., years considered, language, publication status) used as criteria for eligibility, giving rationale. | 4-5 |
| Information sources | 7 | Describe all information sources (e.g., databases with dates of coverage, contact with study authors to identify additional studies) in the search and date last searched. | 5-7 |
| Search | 8 | Present full electronic search strategy for at least one database, including any limits used, such that it could be repeated. | 4-5 |
| Study selection | 9 | State the process for selecting studies (i.e., screening, eligibility, included in systematic review, and, if applicable, included in the meta-analysis). | 4-5 |
| Data collection process | 10 | Describe method of data extraction from reports (e.g., piloted forms, independently, in duplicate) and any processes for obtaining and confirming data from investigators. | 5 |
| Data items | 11 | List and define all variables for which data were sought (e.g., PICOS, funding sources) and any assumptions and simplifications made. | 6-7 |
| Risk of bias in individual studies | 12 | Describe methods used for assessing risk of bias of individual studies (including specification of whether this was done at the study or outcome level), and how this information is to be used in any data synthesis. | 7 |
| Summary measures | 13 | State the principal summary measures (e.g., risk ratio, difference in means). | 6 |
| Synthesis of results | 14 | Describe the methods of handling data and combining results of studies, if done, including measures of consistency (e.g., I^2^) for each meta-analysis. | 5-6 |

| **Section/topic** | **#** | **Checklist item** | **Reported on page #** |
| --- | --- | --- | --- |
| Risk of bias across studies | 15 | Specify any assessment of risk of bias that may affect the cumulative evidence (e.g., publication bias, selective reporting within studies). | N/A |
| Additional analyses | 16 | Describe methods of additional analyses (e.g., sensitivity or subgroup analyses, meta-regression), if done, indicating which were pre-specified. | 7 |
| **RESULTS** | | |  |
| Study selection | 17 | Give numbers of studies screened, assessed for eligibility, and included in the review, with reasons for exclusions at each stage, ideally with a flow diagram. | 8; fig1 |
| Study characteristics | 18 | For each study, present characteristics for which data were extracted (e.g., study size, PICOS, follow-up period) and provide the citations. | 5-6, suppl. |
| Risk of bias within studies | 19 | Present data on risk of bias of each study and, if available, any outcome level assessment (see item 12). | N/A |
| Results of individual studies | 20 | For all outcomes considered (benefits or harms), present, for each study: (a) simple summary data for each intervention group (b) effect estimates and confidence intervals, ideally with a forest plot. | Fig 1, fig2 |
| Synthesis of results | 21 | Present results of each meta-analysis done, including confidence intervals and measures of consistency. | N/A |
| Risk of bias across studies | 22 | Present results of any assessment of risk of bias across studies (see Item 15). | N/A |
| Additional analysis | 23 | Give results of additional analyses, if done (e.g., sensitivity or subgroup analyses, meta-regression [see Item 16]). | N/A |
| **DISCUSSION** | | |  |
| Summary of evidence | 24 | Summarize the main findings including the strength of evidence for each main outcome; consider their relevance to key groups (e.g., healthcare providers, users, and policy makers). | 12-13 |
| Limitations | 25 | Discuss limitations at study and outcome level (e.g., risk of bias), and at review-level (e.g., incomplete retrieval of identified research, reporting bias). | 10-13 |
| Conclusions | 26 | Provide a general interpretation of the results in the context of other evidence, and implications for future research. | 10-12 |
| **FUNDING** | | |  |
| Funding | 27 | Describe sources of funding for the systematic review and other support (e.g., supply of data); role of funders for the systematic review. | Direct upload |

Elements of this paper have been previously published in the Emory University Electronic Theses and Dissertations repository, located here: <http://pid.emory.edu/ark:/25593/bqkc2>.

1. Special thanks to John Harris for supplying the full series of data. [↑](#footnote-ref-2)
